# Active peptides of TSP-1 inhibit retinal angiogenesis through the CD36 pathway in a rat model of choroidal neovascularization

**DOI:** 10.1371/journal.pone.0325661

**Published:** 2025-06-20

**Authors:** Yadi Li, Aiping Deng, Kangwei Jiao, Jie Yan, Wandong Zuo, Yujie Dong, Wenrong Xu, Yuting Li, Chunming Guo, Maorong Chen, Run Tian, Zhulin Hu

**Affiliations:** 1 Yunnan University, Kunming, Yunnan, China; 2 Key Laboratory of Yunnan Province, Yunnan Eye Institute, Affiliated Hospital of Yunnan University, Kunming, Yunnan, China; 3 Key Laboratory of Yunnan Province for the Prevention and Treatment of Ophthalmology, Kunming, Yunnan, China; 4 School of Clinical Medicine, Dali University, Dali, Yunnan Province, China; 5 Yunnan Key Laboratory of Cell Metabolism and Disease, Center for Life Sciences, School of Life Sciences, Yunnan University, Kunming, Yunnan, China; PearlsInMires, KOREA, REPUBLIC OF

## Abstract

**Background:**

Choroidal neovascularization (CNV) is a key manifestation of intraocular neovascularization, and it is considered one of the main causes of blindness in ophthalmology. Additionally, multiple anti-vascular endothelial growth factor (VEGF) drugs have been used as first-line treatment for CNV. However, several issues posed challenges to the anti-VEGF drugs, which were mainly composed of short duration of action, requirement for repeated injections, and complications. Thrombospondin-1 (TSP-1) is an endogenous protein that was found to regulate multiple biological processes within the body, and it has been proven to exhibit an inhibitory effect on neovascularization. Besides, the function of TSP-1 during the inhibition of neovascularization was currently considered to mainly focus on its type Ⅰ repeats (TSRs), which was attributed to the large molecular weight, complex structure, and possible unknown functions of TSP-1. Therefore, TSRs can be applied as targets and research directions for the further development and exploration of potential therapeutic drugs.

**Objectives:**

Based on the type I repeats (TSRs) of thrombospondin-1 (TSP-1), amino acid sequences of different lengths were designed and synthesized in this study, named as VR-9 VR-10、VR-11、VR-12、VR-13. The objective was to explore the effects of the above five peptides on angiogenesis in Chori-retinal neovascularization, alongside the screening of the best peptides and the deep exploration into the underlying mechanism, aimed to provide a basis for the development and application of peptide drugs in the treatment of CNV.

**Methods:**

Wound healing, CCK-8, and 5-ethynyl-2′-deoxyuridine (EdU) assays were employed to evaluate the proliferation and migration ability of cells. CRISPR-Cas9 technology was utilized to establish CD36 knockdown cell lines, alongside the conduction of qPCR to verify the efficiency of gene knockdown. The expression levels of VEGF and CD31 in RF/6A cells and rats were assessed by Western blot. Additionally, Hematoxylin and eosin (HE) staining was performed to examine the structural integrity of the rat retina, while Fluorescein Isothiocyanate-Dextran Cardiac Perfusion (FITC) labeling was used to observe the occurrence and development of choroidal neovascularization (CNV).

**Results:**

According to the wound-healing and CCK-8 assays, VR-13 was the most effective in inhibiting the proliferation and migration of endothelial cells. Furthermore, VR-13 peptide effectively inhibited the pathological development of CNV without the detection of retinal toxicity in the rat CNV model.

**Conclusions:**

Overall, it was found that VR-13 exhibit significant effects on the inducing of apoptosis and the inhibition of the progression of angiogenesis by regulating the expression of VEGF and CD31 via CD36 signaling pathway.

## 1. Introduction

Pathological angiogenesis tended to occur in most eye tissues, including the cornea, retina, choroid, and iris, which would typically result in blindness. Choroidal neovascularization (CNV) is a key manifestation of intraocular angiogenesis, which is commonly associated with eye diseases such as diabetic retinopathy, age-related macular degeneration (AMD), retinopathy of prematurity, and ischemic retinal vein occlusion [1]. Additionally, high permeability of the vascular wall tended to result in local bleeding and exudation. With the progression of neovascularization to fibrous hyperplasia, it exhibited damaging effects on the ocular structure and decreased the visual function, which ultimately resulted in severe visual impairment [2]. Therefore, further research on the mechanisms and prevention of ocular neovascularization is in urgent need to provide clinical guidance for its treatment.

To date, multiple anti-vascular endothelial growth factor (VEGF) drugs have been applied in the first-line treatment of ocular angiogenesis. However, the application of anti-VEGF drugs was restricted by several problems, including short duration of action, requirement for repeated injections, complications such as endophthalmitis, lens damage, retinal detachment, high intraocular pressure, and drug resistance, and all the above problems limited the efficacy of anti-VEGF drugs [3–6]. Therefore, the discovery of novel targets, alongside the investigation of effective treatment options, was crucial for the further development of relevant technologies.

The thrombospondin (TSP) family is composed of five members (TSP-1 to TSP-5) that belong to the extracellular matrix, which exhibits significant influences on multiple pathophysiological processes, including wound healing, vascular function, synapse formation, immune response, and inflammation based on the interaction with various cell surface receptors and proteases [7]. Additionally, TSPs could be divided into subgroups A and B, in which the TSP-1 was a member of subgroup A and harbored a TSP-1 repeat (TSR), which exhibited endogenous protective functions in the inhibition of angiogenesis. It was confirmed by previous studies that TSP-1 showed the ability to inhibit angiogenesis based on the induction of endothelial cell apoptosis and mediation of immune regulation under the conditions of corneal diseases, diabetic retinal diseases, and tumors [8–10]. Nevertheless, the synthesis and the efficient delivery of TSP-1 remained to be challenging doe to its large molecular weight and complex structure. Moreover, it has been suggested that the C-terminal region of TSP-1 could be bound to CD47, which could facilitate the cell migration and adhesion, contributing to the promotion of angiogenesis [11]. Nevertheless, the relevant research on the functions of TSP-1 in ocular neovascularization was relatively controversial and remained in the preliminary stage of investigation, and further exploration and development was necessary for the actual application.

The investigation of the inhibition effects of TSP-1 on neovascularization was further focused on its different domains due to the large molecular weight and complex structure of TSP-1. Among the different domains of TSP-1, Thrombospondin type I repeats (TSRs) was confirmed to exhibit endogenous protective functions in inhibiting neovascularization, and this segment was thought to contain a CD36 binding site. Therefore, it was considered as the structural basis in the inhibition of angiogenesis. This study aims to synthesize the active peptide fragment of TSP-1 based on TSRs, alongside the screening of the best peptide fragment in the inhibition of CNV through cell experiments on VR-9, VR-10, VR-11, VR-12 and VR-13, as well as the further exploration into the underlying mechanism, which could provide a basis for the clinical transformation of TSP-1 active fragment peptides.

Previously, peptides of different lengths were synthesized based on TSRs (including VR-9 to VR-13), and it was discovered that VR-10 (peptide of TSP-1) [12] showed inhibitory effects on the endothelial cell proliferation and migration. However, the mechanisms by which the inhibition of angiogenesis was conducted remained to be clarified. Additionally, the actual functions of VR-9, VR-11, VR-12, VR-13, and other peptides remained unclear. Additionally, RF/6A was an established Chori-retinal endothelial cell line, and RF/6A cells were employed as experimental models to fill the knowledge gap mentioned above. It was designed to conduct the test of each TSP-1 peptide fragment by the CCK-8 and wound healing assays, in order to identify the optimal peptide for the inhibition of both proliferation and migration of the endothelial cells. According to the results, all synthesized peptides exhibited significant inhibitory effects on the above factors, among which VR-13 appeared to show the most effective functions. Besides, the CNV model with Sprague-Dawley rats was employed in this study to verify this result, and VR-13 was confirmed to inhibit the progression of angiogenesis by modulating the expression levels of VEGF and CD31. Moreover, the expression of CD36 was suppressed at the cellular level to reveal the precise mechanism by which the TSP-1 peptide inhibited the angiogenesis, which was aimed for the deep exploration of the relationship between TSP-1 peptide and CD36. In conclusion, it could be indicated by the results that the TSP-1 synthetic peptide could effectively inhibit the proliferation and migration of endothelial cells, and VR-13 exhibited the most significant efficacy. Furthermore, the inhibition of angiogenesis might be realized by modulating the levels of VEGF and CD31 through the CD36 signaling pathway.

## 2. Materials and methods

### 2.1. Ethical approval

The animal study protocol was approved by the Institutional Review Board (or Ethics Committee) of Laboratory Animal Welfare Ethics Committee Yunnan University (protocol code: YNU20240727; date of approval: 2024/02/13).

### 2.2. Cell culture

RF/6A cells were obtained from Cell Cook, China. The cells were maintained in minimum essential medium supplemented with 10% fetal Bovine serum (Viva Cell, China) and 1% penicillin-streptomycin solution (Viva Cell, China). Additionally, the samples were cultured in an incubator under the condition of 37 °C and 5% CO_2_.

### 2.3. Cell growth and proliferation

The Cell Counting Kit-8 (CCK-8) from Proteintech (China) and 5-ethynyl-2′-deoxyuridine (EdU)-488™ kit from Beyotime (China) were employed for the assessment of the proliferative viability of RF/6A cell. Additionally, 10^4^ cells were plated in 96-well plates. After 24 h of intervention, according to different groups, CCK-8 detection reagent was added, and the optical density was recorded at 450 nm during the following 4 h. Regarding the EdU assay, 2 × 10^4^ cells were cultured in 24-well plates after 24 h of intervention according to different groups. Firstly, the EdU working solution (20μM) preheated at 37 ° C was combined with medium at the ratio of 1:1. Secondly, the cells were washed three times with (phosphate buffered saline) PBS after the fixing with 4% paraformaldehyde for 15 min. Subsequently, the samples were permeabilized with PBS that contained 0.3% Triton X-100 for 10 min, followed by three times of washing with PBS, after which the EdU detection was conducted. Whereafter, 100 μL Click reaction solution was added to each well and incubated at room temperature in the dark for 30 min, followed by washing again with PBS, the Click reaction solution configuration method was shown in Table 1. Finally, fluorescence detection was performed after nuclear staining by Hoechst 33342. (Hoechst 33342 exhibits excitation/emission: 346 nm/460 nm and azide 488 with excitation/emission: 495 nm/519 nm).

**Table 1:** Click reaction solution configuration method.

Component	Click Reaction Buffer	CuSO_4_	Azide 488	Click Additive Solution	Total volume
	86μl	4μl	0.2μl	10μl	100μl

### 2.4. Wound-healing assay

After the drawing of 3 parallel lines at the external bottom surface of a 6-well plate, 4 × 10^5^ RF/6A cells were seeded in it and incubated to a density of 80–90%. Next, scratches perpendicular to the drawn lines were made at the bottom of each well by a 200 μL pipette tip. Subsequently, the wells were rinsed three times with PBS to eliminate the floating cells. Whereafter, the medium was replaced with the serum that contained 1% fetal bovine. The wound healing process was assessed at the time point of 0 h and 24 h, alongside the acquirement of microscope images at 10× for the record and statistical analysis of the scratch distance. Additionally, the scratch healing rate was calculated as follows:


$$Scratch\_healing\_rate=\frac{0h\_scratch\_distance-24h\_scratch\_distance}{0h\_scratch\_distance}100\;\%$$ (1)

### 2.5. Animals

Male Sprague-Dawley rats (aged between 6 and 8 weeks) were sourced from Kunming Medical University and maintained in pathogen-free conditions, and the CNV model was induced by subretinal injection of Matrigel (Corning, USA). All the rats were anesthetized by 2% sodium pentobarbital with a dose of 50 mg/kg. VR-13 (1 μL, 1 mg/mL) was then administered intravitreally into the right eye of the rats via a micro syringe. Besides, all rats were sacrificed by neck removal after anesthesia.

### 2.6. Hematoxylin and eosin staining

The extracted eyeball was cut into thick sections of 8–10 μm. These sections were fixed with tissue fixative for 15 min, washed with running water, followed by incubation in a hematoxylin staining solution for 3–5 min. The solution was differentiated, which resulted in the returning of the blue staining solution to blue. Following each step, the sections were rinsed with PBS (3–5 min), dehydrated in 95% alcohol (1 min), stained with eosin (15 s), and then immersed in absolute ethanol (I, II, III), n-butanol (I, II), and xylene (I, II) for 2 min each. Additionally, the samples were mounted with neutral gum after becoming transparent.

### 2.7. CD36 knockdown in RF/6A cells

After the increasing of adherent confluence rate of cell growth to 50–80%, the cells were collected and then washed twice with Dulbecco’s PBS, followed by digestion with 1 mL of 0.25% tryp-sin-ethylenediaminetetraacetic acid in the incubator for 3 min, termination of the digestion with the addition of 5 mL of complete medium. Subsequently, the super-natant was discarded by the centrifugation at 300 g for 3 min, and the samples were re-suspended in 1 mL of Dulbecco’s PBS. Additionally, 2 × 10^5^ cells/group were collected in the experiment: one knockdown group and one control group. After 300 g centrifugation for 3 min, 5 μL of Buffer R was added to each group, while 6 μL of CRIPSR-Cas9/gRNA complex and 6 μL of Buffer R was added to the knockdown group and the control group, respectively. After the electroporation, based on the Neon™ transfection system, the genomic DNA extraction was performed, followed by the amplification of the CD36 fragment for sequencing. Besides, RNA and primers were bio-synthesized by Genechem (Shanghai Co., Ltd), and the sequence information was shown in Table 2.

**Table 2:** sgRNA and primer sequences.

Target	Seq Name	Target Seq (5’-3’)
*CD36*	*CD36-*KO-sg1	ATGGGCTGTGACCGCAACTG
	*CD36-*KO-sg2	GGAGGTATTCTAATGCCAGT
	*CD36-*KO-sg5	CTTTGATGTGCAAAATCCAC
	*CD36-*KO-sg6	AACATTCAAGTTAAGCAAAG
	*CD36*-KO-sg1/2-F1	GGGAGGTGGACTGAGTAAGTCA
	*CD36*-KO-sg1/2-R1	AAGATAGCAATGGAGTCGTGT
	*CD36*-KO-sg5/6-F1	CGGTCACTCTAAAGCTGCCA
	*CD36*-KO-sg5/6-R1	AGGATGCTACACAGATAGGAAAACT

### 2.8. Quantitative Real-Time Polymerase Chain Reaction (qPCR)

Total RNA extraction from RF/6A cells was isolated by the TRIzol reagent (ThermoFisher Scientific). The mRNA concentration was determined by SYBR Green qPCR (Roche, Basel, Switzerland) on a Light Cycler® 480 System, in which the qPCR procedure was composed of an initial denaturation step at 95 °C for 10 min, within 40 cycles of amplification (95 °C for 15 s and 60 °C for 30 s). The primers used in this study were synthesized and designed by Takara Bio Inc., and the sequences were as follows:

CD36 (forward): 5’-GAAACCCACACTAACGAGTTCATCA-3’,

CD36 (reverse primer): 5’-TGCCTCACAAAGCTTGCCATA-3’,

β-actin (forward primer): 5’-GCTGTGCTATGTTGCTCTAG-3’,

β-actin (reverse primer): 5’-CGCTCGTTGCCAATAGTG-3’.

### 2.9. Western blot analysis

Western blot analysis was employed in this study for the assessment of the expression levels of proteins. Total protein was extracted with Radioimmunoprecipitation (RIPA) buffer (Solarbio, China) that contained phenylmethanesulfonyl fluoride (PMSF), followed by separation by 10% sodium dodecyl sulfate–polyacrylamide gel electrophoresis (SDS-PAGE, Yazyme, China). Additionally, the constant pressure of 80 V was used for 40–50 min during the electrophoresis of the proteins in compression gel. Besides, the voltage was adjusted to 120 V after the entering of proteins to the separating gel. Subsequently, proteins were transferred onto a polyvinylidene difluoride (PVDF) membrane (Millipore, USA), and the operation time was determined according to the molecular weight of the protein (VEGF-55 min, CD31-91 min). The membrane was blocked with 5% non-fat milk for 2 h and incubated with primary antibodies overnight at 4 °C, and the primary antibody was diluted with Tris-Buffered Saline with Tween 20 (TBST) following the instructions. Subsequently, the samples were washed three times with TBST, incubated with the corresponding secondary antibody for 90 min at room temperature, and washed three times with TBST. Besides, Electrochemiluminescence (Invitrogen iBright™ FL1500) was used for the visualization of proteins, followed by the imaging analysis of the experimental results.

The antibodies utilized in this study were composed of anti-VEGF (Proteintech, China, 1:1000), anti-CD31 (Immunoway, China, 1:1000), and anti-β-actin (Proteintech, China, 1:10000).

### 2.10. Fluorescein isothiocyanate-dextran cardiac perfusion

During this experiment, the rats were anesthetized, alongside the exposure of the heart, and the intracardiac fluorescein per-fusion was carried out with fluorescein isothiocyanate-dextran solution (Sigma, Germany) until the lips and limbs of the rats appeared to be fluorescent yellow. Subsequently, the rats were euthanized, and their eyeballs were removed, alongside the fixing of the samples in the FAS eyeball fixation solution (Servicebio, China). Under the microscope, the cornea, lens, and vitreous humor were excised, and the retina pigmented epithelium (RPE)-choroid-scleral complex was cut into 5–6 radial patterns, followed by the fixing of the samples. Additionally, confocal microscopy was employed in the observation of the occurrence and development of CNV.

### 2.11. Statistical analysis

All the experiments in this study were repeated for at least three times. Additionally, all the experimental data were expressed by Mean ± standard deviation (Mean ± SEM), and the statistical analysis and statistical graph were performed by GraphPad Prism software (GraphPad, Boston, MA, USA). Independent sample T-test was employed in the comparison of the mean difference between the two independent samples (such as control group and experimental group), paired T-test was used to compare the measurement data at different time points within the same group. Besides, one-way ANOVA was employed in the comparison of the measurement data between multiple groups, and multivariate ANOVA was used to compare the measurement data between different groups. Moreover,·Shapiro-Wilk test and Levene test were performed on all the data. Notably, the corrected t-test (Welch method) was used under the condition that the variance was not uniform. When the data failed to meet the distribution, the non-parametric test (Kruskal-Wallis H) would be used. *, *P* ＜ 0.05; **, *P* ＜ 0.01; ***, *P* ＜ 0.001; ****, *P* ＜ 0.0001.

## 3. Results

### 3.1. TSP-1 peptides (VR-9, VR-10, VR-11, VR-12, and VR-13) inhibit the proliferation and migration of RF/6A cells

All the synthetic peptides at different concentrations were tested by the CCK-8 and wound-healing assays, which was aimed to determine the optimal TSP-1 synthetic peptide in the inhibition of endothelial cell proliferation and migration.

**Fig 1:**
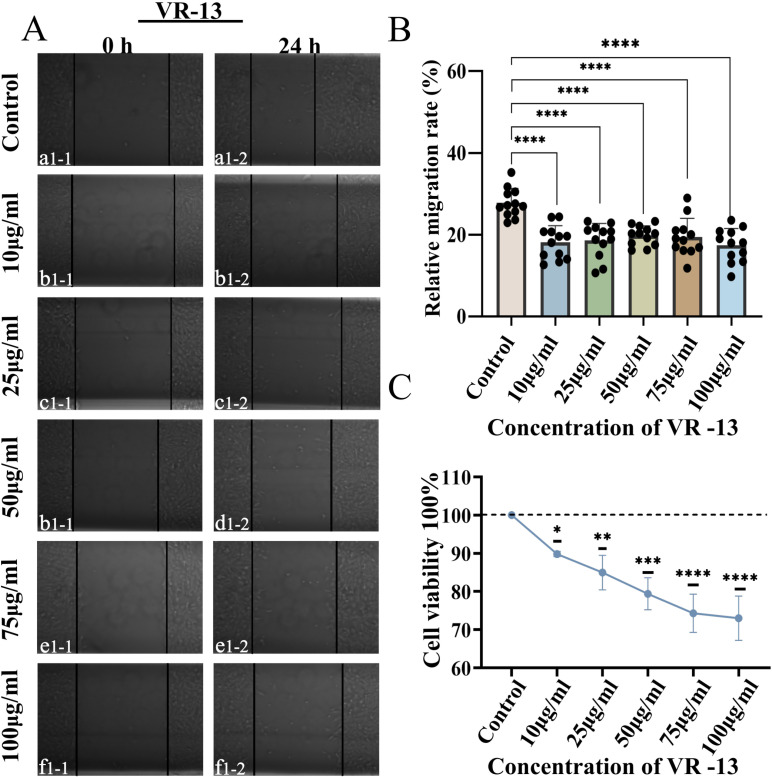
Different concentrations of thrombospondin-1 (TSP-1)-derived peptides (VR-13) inhibited the proliferation and migration of RF/6A. A wound-healing assay was employed in the assessment of the inhibitory effects of different concentrations of VR-13 on the proliferation and migration of RF/6A cells, n = 12. VR-13 inhibited cell proliferation and migration with the concentrations of 10 μg/mL, 25 μg/mL, 50 μg/mL, 75 μg/mL, and 100 μg/mL (A, B). CCK-8 cell viability assay showed that the inhibitory effects of VR-13 peptide on cell proliferation exhibited an increasing trend with the increase of concentrations, n = 3 (C) (*, *P* < 0.05; **, *P* < 0.01; ***, *P* < 0.001; ****, *P* < 0.0001).

It could be demonstrated by the wound-healing assay that VR-9 to VR-13 inhibited RF/6A migration in a manner that was dependent on their concentrations (S1 Fig and Fig 1A and 1B). Additionally, both VR-9 and VR-11 exhibited the ability to inhibit the cell proliferation and migration with the concentration of 75 μg/mL and 100 μg/mL. Besides, VR-11 suppressed RF/6A cell migration with the concentration of 50 μg/mL (S1 Fig A, B, and D). In addition, VR-10 inhibited the RF/6A migration at all concentrations except for 10 μg/mL (S1 Fig A and C). Notably, VR-12 and VR-13 exhibited the ability to inhibit the cell migration with all concentrations (S1 Fig A and E and Fig 1A and 1B). Overall, VR-13 showed the most significant inhibitory effects on cell proliferation and migration among all the groups (S1 Fig F). Furthermore, it was indicated by the CCK-8 cell viability assay that each TSP-1 peptide exhibited an inhibitory effect on cell proliferation increased with the increase of the concentration, among which VR-13 showed the best inhibitory effects (Fig 1C and S1 Fig G). Overall, the need for further research on VR-13 regarding its potential in the inhibition of retinal angiogenesis was emphasized by the above findings.

### 3.2. VR-13 peptide significantly inhibits VEGF-induced cell proliferation and migration

An in-depth investigation was conducted into the inhibitory effects of VR-13 on cell proliferation and migration, which was aimed at the exploration of the underlying mechanisms. Firstly, the pathological process of angiogenesis was simulated in this study, and exogenous VEGF was employed to stimulate the endothelial cell proliferation and migration. Subsequently, VR-13 was added into the samples under this condition, in order to evaluate the ability of VR-13 to restrain the atypical cell proliferation and migration.

**Fig 2:**
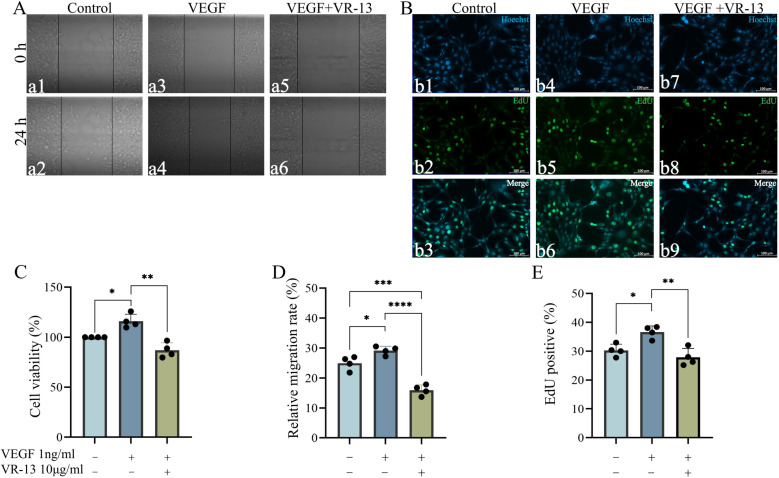
VR-13 peptide inhibited the cell proliferation and migration caused by exogenously supplied vascular endothelial growth factor (VEGF). CCK-8, wound-healing, and 5-ethynyl-2′-deoxyuridine (EdU) assays(n = 4) were used to evaluate inhibition of proliferation and migration of RF/6A cells, in which the RF/6A cells were incubated with 1 ng/mL of exogenously supplied VEGF and 10 μg/mL of VR-13 peptide for 24 h. Exogenous VEGF could significantly stimulate the proliferation and migration of RF/6A, while VR-13 exhibited the ability to inhibit this function. (*, *P* ＜ 0.05; **, *P* ＜ 0.01; ***, *P* ＜ 0.001; ****, *P* ＜ 0.0001).

Additionally, the CCK-8 assay with different concentrations of recombinant human VEGF165 protein (EC_50 _= 0.3–3.75 ng/mL) was employed to examine the RF/6A cell proliferation, and the optimal concentration for inducing significant proliferation of RF/6A was determined as 1 ng/mL (S2 Fig A). CCK-8 and EdU assays were conducted to evaluate the cellular proliferation after a 24 h exposure to exogenous VEGF, which was amined to analyze the impact of 10 μg/mL VR-13 peptide on RF/6A cell proliferation induced through exogenous VEGF. It was indicated by the findings that 10 μg/mL of the VR-13 peptide could suppress the RF/6A cell proliferation stimulated by 1 ng/mL of exogenously supplied VEGF (Fig 2B, 2C and 2E, ). Besides, it was revealed by the wound-healing assay that RF/6A cells exposed to VEGF exhibited strong cell migration ability, alongside the significant inhibitory effects of VR-13 on VEGF-induced cell migration (Fig 2A and 2D ).

### 3.3. VR-13 downregulates VEGF and CD31 in RF/6A cells and Induces apoptosis

Angiogenesis was typically accompanied by the apoptosis of endothelial cells, which could be attributed to the association of angiogenesis with the expression of VEGF. Western blotting was employed in the analysis of the expression levels of angiogenesis-related proteins to clarify the mechanisms by which VR-13 inhibited the cell proliferation and migration, alongside the determination of the extent of cell apoptosis.

**Fig 3:**
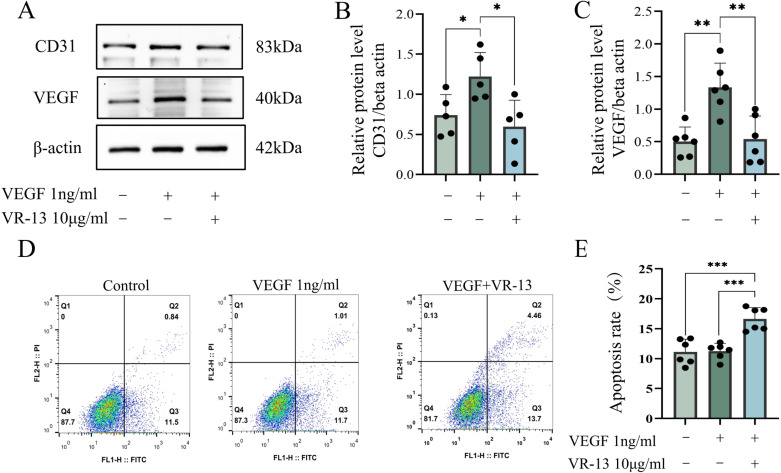
In RF/6A cells, the VR-13 peptide inhibited the neovascularization by regulating angiogenesis-related protein levels, such as those of VEGF (n = 6) and CD31 (n = 5), alongside the inducing of cell apoptosis. (A–C) The expression of angiogenesis-related proteins in each group was examined by western blotting. (D, E) The total apoptosis rate in each group was calculated by flow cytometry, n = 6 (*, *P* ＜ 0.05; **, *P* ＜ 0.01; ***, *P* ＜ 0.001; ****, *P* ＜ 0.0001).

The VEGF and CD31 within RF/6A cells were significantly upregulated after treatment with exogenous VEGF (1 ng/mL) (Fig 3A-C), whereas VEGF and CD31 were downregulated after the intervention with VR-13 with a concentration of 10 μg/mL. Moreover, it was found by flow cytometry that incubation with exogenous VEGF didn’t exhibit a connection with apoptosis. However, 10 μg/mL of the VR-13 peptide could induce apoptosis in RF/6A cells after treatment with 1 ng/mL VEGF (Fig 3D and 3E).

### 3.4. VR-13 peptide downregulates VEGF and CD31 in a rat matrigel CNV model

**Fig 4:**
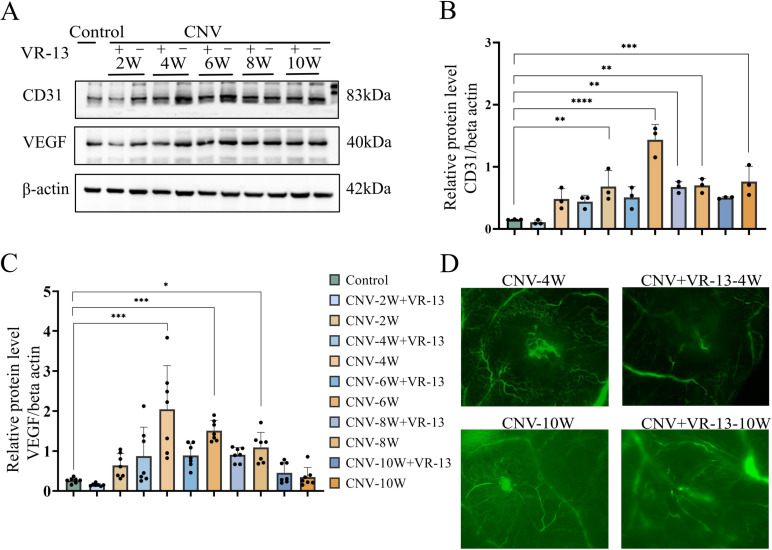
In a rat Matrigel CNV model, the VR-13 peptide exhibited inhibitory effects on the CNV pathological progression by regulating the VEGF and CD31 levels. (A–C) Western blot for the detection of the VEGF (n = 7) and CD31 (n = 3) levels in each group (D) Fluorescein isothiocyanate-dextran angiography for the observation of the pattern of the retinal vasculature n = 3. (*, *P* ＜ 0.05; **, *P* ＜ 0.01; ***, *P* ＜ 0.001; ****, *P* ＜ 0.0001).

The SD rat CNV model was constructed by subretinal injection of Matrigel and monitored for 2, 4, 6, 8, and 10 weeks in order to comprehensively characterize the underlying mechanisms of the inhibitory effects of VR-13 on angiogenesis. Subsequently, western blot analysis was employed in the evaluation of the expression levels of proteins involved in angiogenesis. Compared with the control rats, no noticeable differences in angiogenesis-related proteins were observed in the retinal tissue across each group at 2 weeks, and significant differences occurred at 4 weeks, which could suggest that the peak effects of VR-13 on this model were at 4 weeks. Additionally, the levels of VEGF and CD31 proteins in the CNV group showed a progressively increasing trend (Fig 4A-C). Besides, the expression of VEGF and CD31 reached a peak, followed by a gradual decline after 4 weeks. Furthermore, at 2, 4, 6, and 8 weeks, the expression of the above factors in the VR-13 peptide intervention group was consistently lower compared with that in the same group. Overall, it could be suggested by these results that VR-13 could sustainably inhibit angiogenesis *in vivo*.

### 3.5. VR-13 peptide effectively inhibits the pathological development of CNV without causing retinal toxicity

The rat Matrigel CNV model was observed over a period of 2, 4, 6, 8, and 10 weeks, in order to characterize the effects and duration of VR-13 in the inhibition of neovascularization *in vivo*. Additionally, the peak and end times of the model were selected for observation. FITC-Dextran angiography was employed in the assessment of the area of CNV leakage, and it was markedly reduced in the VR-13-treated group versus the CNV group at 4 and 10 weeks (Fig 4D). In addition, it was revealed by hematoxylin and eosin staining that no significant pathological alterations were observed in the retinal structure as model group after the intervention of VR-13 versus the normal eyes (S2 Fig C).

### 3.6. VR-13 inhibits the endothelial cells proliferation and migration as downregulates VEGF and CD31 via the CD36 pathway

**Fig 5:**
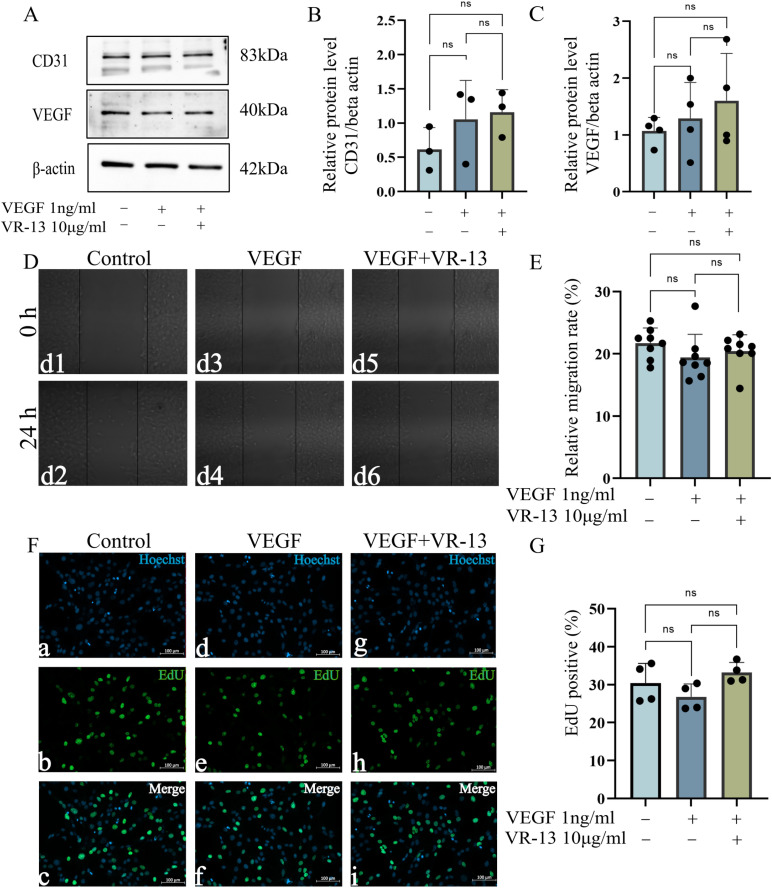
VR-13 peptide was bound to CD36 to regulate endothelial cell proliferation and migration, alongside the inhibition of angiogenesis by controlling the levels of the angiogenesis-related proteins VEGF and CD31. (A–C) Western blots for the detection of the VEGF (n = 4) and CD31(n = 3) levels; (D–G) wound-healing (n = 8) and EdU assays(n = 4) for the evaluation of the inhibitory ability of VR-13 on cell proliferation and migration in CD36 knockdown cells. (*, *P* ＜ 0.05; **, *P* ＜ 0.01; ***, *P* ＜ 0.001; ****, *P* ＜ 0.0001).

A *CD36* knockdown cell line was constructed by CRISPR/Cas9 technology, and the knockdown was verified by qPCR (S2 Fig B). Exogenous VEGF was employed to induce the proliferation of these knockdown cells with the treatment of VR-13, in order to further verify the functions of *CD36* in the inhibitory effects of VR-13 peptide on cell proliferation and migration. Besides, it was revealed by wound-healing and EdU experiments that the inhibitory effects of VR-13 on cell proliferation and migration were reversed (Fig 5D-G), and it was indicated by Western blotting that the downregulation of VEGF and CD31 caused by VR-13 was also reversed (Fig 5A-C)

## 4. Discussion

Although the intravitreal injection of anti-VEGF is an effective treatment option in the prevention of vision loss or improvement of vision in patients with CNV [2], it is relatively expensive and has been shown to result in various complications, including endophthalmitis, cataracts, retinal artery occlusion, or other complications [13–15]. TSP-1 was an endogenous angiogenesis inhibitor [16], and it was confirmed to exhibit neovascularization inhibition effects in tumor, ischemia, and hypoxia models [17–19]. Additionally, TSP-1 could interact with various ligands, including extracellular matrix structural components, matricellular proteins, growth factors, receptors, proteases, and cytokines, and it could be divided into six domains. Among these domains, Type I repeats (TSRs) might interact with matrix metallopeptidase-2 (MMP-2), MMP-9, CD36, β1 integrins, latent transforming growth factor‑β, and CD148 [20,21]. Besides, TSRs were considered the structural basis of the TSP-1-mediated inhibition of endothelial cell proliferation, induction of endothelial cell apoptosis, and inhibition of angiogenesis [22,23]. Therefore, corresponding derivatives based on the structure of type I repeats were synthesized by researchers such as ABT-526 (N-Ac-Sar-Gly-Val-D-Ile-Thr-Nva-Ile-Arg-Pro-NHEt) and ABT-510 (Ac-Sar-Gly-Val-Dallolle-Thr-Nva-Ile-Arg-Pro-NHEt), which were anti-angiogenic nonapeptides. Additionally, it was revealed by *in vivo* studies that both ABT-526 and ABT-510 could slow down the tumor growth in syngeneic and xenograft mouse models, alongside the increase of the apoptotic index of endothelial cells, such as TSP-1. Besides, ABT-510 was analyzed in many studies, which was primarily synthesized based on the first repeat of TSRs (GVITRIR), and it could inhibit VEGF-induced angiogenesis [24–26]. Moreover, the second and third TSR repeats (CSVTCG and VTCGVITRIR, respectively) exhibited crucial functions in the inhibition of the endothelial cell proliferation and migration. Therefore, TSP-1 polypeptides of different lengths from the three TSR repeats were synthesized by our team: VR-9, VR-10, VR 11, VR-12, and VR-13. Due to the fact that endogenous inhibitors showed little inherent toxicity, it could be hypothesized that these peptides would possess the anti-angiogenic effects of TSP-1. Therefore, cell and animal experiments were employed in the validation of the samples. Additionally, CCK-8 and wound-healing assays were used to verify the effects of TSP-1 polypeptides with different concentrations. Under physiological conditions, VR-9, VR-10, VR-11, VR-12, and VR-13 all exhibited the inhibitory effects on endothelial cell proliferation and migration, among which VR-13 showed the strongest ability.

CNV was essentially an angiogenesis process that primarily occurred in the matrix surrounding blood vessels [27]. The three important factors in angiogenesis were endothelial cell proliferation and migration, extracellular matrix degradation, and cytokine release or production. Additionally, endothelial cells were in a stationary state under normal conditions, and a dynamic balance existed between pro-angiogenic and anti-angiogenic factors. However, the balance between pro- and anti-angiogenic factors would be disrupted with the occurrence of hypoxia, and hypoxia-inducible factor-1 (HIF-1) contributed to the secretion of pro-angiogenic factors in RPE cells such as VEGF, platelet-derived growth factor, and fibroblast growth factor, among which VEGF could promote endothelial cell proliferation, migration, and angiogenesis [28,29]. According to the above findings, angiogenesis was primarily initiated by pro-angiogenic factors.

RF/6A cells were treated with exogenous VEGF to mimic pathological angiogenesis, and it was found that 1 ng/mL of recombinant human VEGF165 could significantly stimulate the endothelial cell proliferation, which also exhibited a strong migration ability. With the addition of VR-13 into the cell culture, the inhibitory effects of VR-13 on angiogenesis were observed under the aforementioned pathologies. Although the inhibitory functions of VR-13 under pathological conditions were confirmed by the above results, the specific mechanisms of the action of VR-13 were required to be further explored.

Firstly, it was found that VR-13 could downregulate the expression of VEGF and CD31 and aggravate the RF/6A cell apoptosis. Additionally, it was suggested by previous studies that TSP-1 might inhibit angiogenesis by antagonizing the VEGF–VEGFR2 signaling pathway, and VEGF-A exhibited crucial functions during the development of CNV in AMD [29,30]. VEGF-A was an important angiogenesis regulator, and its overexpression or abnormal secretion was considered to be related to angiogenesis, tumor growth, cell proliferation, and immune regulation. Besides, the pathogenic effects of VEGF-A could be predominantly attributed to its influence on vascular permeability and angiogenesis [31]. Given the dominant functions of VEGF-A in the regulation of angiogenesis, it was referred to as VEGF in most studies.

VEGF-A showed multiple functions in various physiological and pathological processes [30,32], which was composed of physiological and pathological angiogenesis, bone morphogenesis, corpus luteum development, pregnancy, and vascular permeability. Additionally, it could stimulate the endothelial cell proliferation through the PLCγ-PKC-MAPK signaling pathway by binding to VEGFR2. Moreover, knockout mouse models have been shown to be connected with the development of systemic vascular lesions, including bleeding, validation, and microcirculation disorders at the age of 25 weeks. Under this condition, VEGF was confirmed to drive the maintenance of endothelial cell homeostasis and survival, which could prevent these initially inevitable outcomes [33].

CD31 [34] was expressed in endothelial cells and localized to their tight junctions, which could serve as the mechanical sensors of endothelial cells and the regulators of vascular permeability. Additionally, CD31 inhibitors could block the angiogenic ability of endothelial cells, as well as tumor-induced angiogenesis [35–39]. Notably, impaired tumor angiogenesis was also observed in CD31 knockout mice. Based on the above research, VEGF was involved in angiogenesis by regulating endothelial cell survival and vascular permeability. Besides, CD31 was an endothelial cell biomarker [40], and it might be connected with the cell migration and angiogenesis.

Subsequently, a rat model of CNV was employed in this study, and the results were similar to those mentioned above. Additionally, it was observed that VR-13-mediated downregulation of VEGF levels was gradually reduced with the extending of the observation time to 8 weeks, with no observation of significant difference in VEGF levels between the CNV and VR-13 intervention groups. At week 4, the peak of VEGF expression occurred in the CNV group, and the CNV leakage area in the VR-13 intervention group was relatively smaller versus the CNV group. After 10 weeks, no significant difference was observed in VEGF levels between the two groups, and the CNV leakage area in the VR-13 group was still relatively smaller versus the CNV group. Regarding the CNV rat model, it was observed that the inhibition of neovascularization caused by the VR-13 polypeptide lasted for 6 weeks, during which the retinal toxicity caused by VR-13 was not observed. However, only one model was used during this experiment, and the studies were focused on only male rats, which might be one of the main limitations of this study.

It was reported by previous studies [10,41] that TSP-1 that was bound to *CD36* could inhibit angiogenesis in microvascular endothelial cells by promoting endothelial cell apoptosis and inhibiting nitric oxide signaling. Additionally, *CD36* was a multifunctional membrane glycoprotein (scavenger receptor type B) m which was presented in various cells in different tissues and organs [42]. It was found in previous preclinical studies that peptide mimetics based on one of the TSP-1 and *CD36* binding sites showed anti-angiogenic and anti-tumor activities [18]. Besides, the above findings were consistent with the results of our previous study that VR-10 could mediate apoptosis, inflammation, and autophagy by binding to CD36 [43]. Nevertheless, the validation with human experiments was lacking in their findings. VR-13 was derived from a type I repeat of the *CD36* binding site. It was hypothesized that the inhibitory effects on angiogenesis were closely related to *CD36*, and a *CD36* knockdown cell line was constructed to further confirm the inhibitory effects of VR-13 on the proliferation and migration of endothelial cells, alongside the levels of VEGF and CD31 angiogenesis-related proteins. It could be revealed by the results that the effects of VR-13 were reversed after *CD36* knockdown, which was in agreement with the previous findings that TSP-1 would interact with *CD36* to regulate angiogenesis. As a peptide derived from TSP-1, VR-13 was suggested to mediate angiogenesis by binding to *CD36* according to these results, but the specific mechanisms remained unclear. In this study, peptides with low molecular weight were synthesized, and it was demonstrated that these peptides showed inhibitory effects on endothelial cell proliferation and migration, as well as angiogenesis in animal models. In future experiments, it was necessary to further clarify the specific connection between VR-13 and *CD36*, in order to understand the mechanisms by which it inhibited angiogenesis and induced apoptosis.

Low molecular weight peptides were synthesized in this study, and it was demonstrated that these peptides exhibited inhibitory effects on endothelial cell proliferation, migration, and angiogenesis in an animal model. Additionally, these peptides exhibited remarkable effects without driving retinal toxicity, which could provide fundamental research for the future application of VR-13. However, the molecular conformation of VR-13 remained unclear, and its action site required to be further verified. Therefore, it was necessary to clarify the specific mechanism by which VR-13 inhibited angiogenesis and induced apoptosis through *CD36* in future studies, alongside the clarification of its molecular conformation and action site.

## 5. Conclusions

In this study, it was found that VR-13 exhibited the most significant inhibitory effects on the proliferation and migration of endothelial cells among the five TSP-1-derived peptides of different lengths (VR-9, VR-10, VR-11, VR-12, VR-13). Additionally, the pathological progression of neovascularization was inhibited by VR-13 based on the downregulation of the angiogenesis-related proteins VEGF and CD31 in both RF/6A cells and rat model of CNV, and this effect was confirmed to occur via the *CD36* signaling pathway.

## Supporting information

S1 Fig Different concentrations of thrombospondin-1 (TSP-1)-derived peptides (VR-9, VR-10, VR-11, VR-12, and VR-13) inhibit the proliferation and migration of RF/6A. Wound-healing assay was used to detect the inhibitory effect of different concentrations of TSP-1 peptides (VR-9 to VR-12) on the proliferation and migration of RF/6A cells (a–e). VR-9 inhibited cell proliferation and migration at concentrations of 75 μg/mL and 100 μg/mL; VR-10 at concentrations of 25 μg/mL, 50 μg/mL, 75 μg/mL, and 100 μg/mL; VR-11 at concentrations of 50 μg/mL, 75 μg/mL, and 100 μg/mL; VR-12 inhibited cell proliferation and migration at all concentrations. The VR-13 inhibitory effect on cell proliferation and migration was the most significant among all TSP-1 peptide (f). CCK-8 assay showed that the inhibitory effect of each TSP-1 peptide on cell proliferation increased with increasing concentrations, with VR-13 showing the strongest inhibitory effect (g). (*, *P *< 0.05; **, *P *< 0.01; ***, *P * < 0.001; ****, *P * < 0.0001).
(TIF)

S2 Fig CCK-8 assay was used to detect the effect of different concentrations of recombinant human VEGF165 on the proliferation of RF/6A cells (a). CD36 knockdown verification by quantitative real-time polymerase chain reaction (qPCR) (b). Hematoxylin and eosin staining to observe the retinal architecture (c). (*, *P * < 0.05; **, *P * < 0.01; ***, *P * < 0.001; ****, *P * < 0.0001).
(TIF)

S1 Data Minimal Data. (ZIP)

S3 Fig Raw images. (PDF)
