# Supplementary figures and images for: Active peptides of TSP-1 inhibit retinal angiogenesis through the CD36 pathway in a rat model of choroidal neovascularization
Source: PLoS One. 2025 Jun 20;20(6):e0325661. doi: 10.1371/journal.pone.0325661 (PMC12180637; doi:10.1371/journal.pone.0325661)

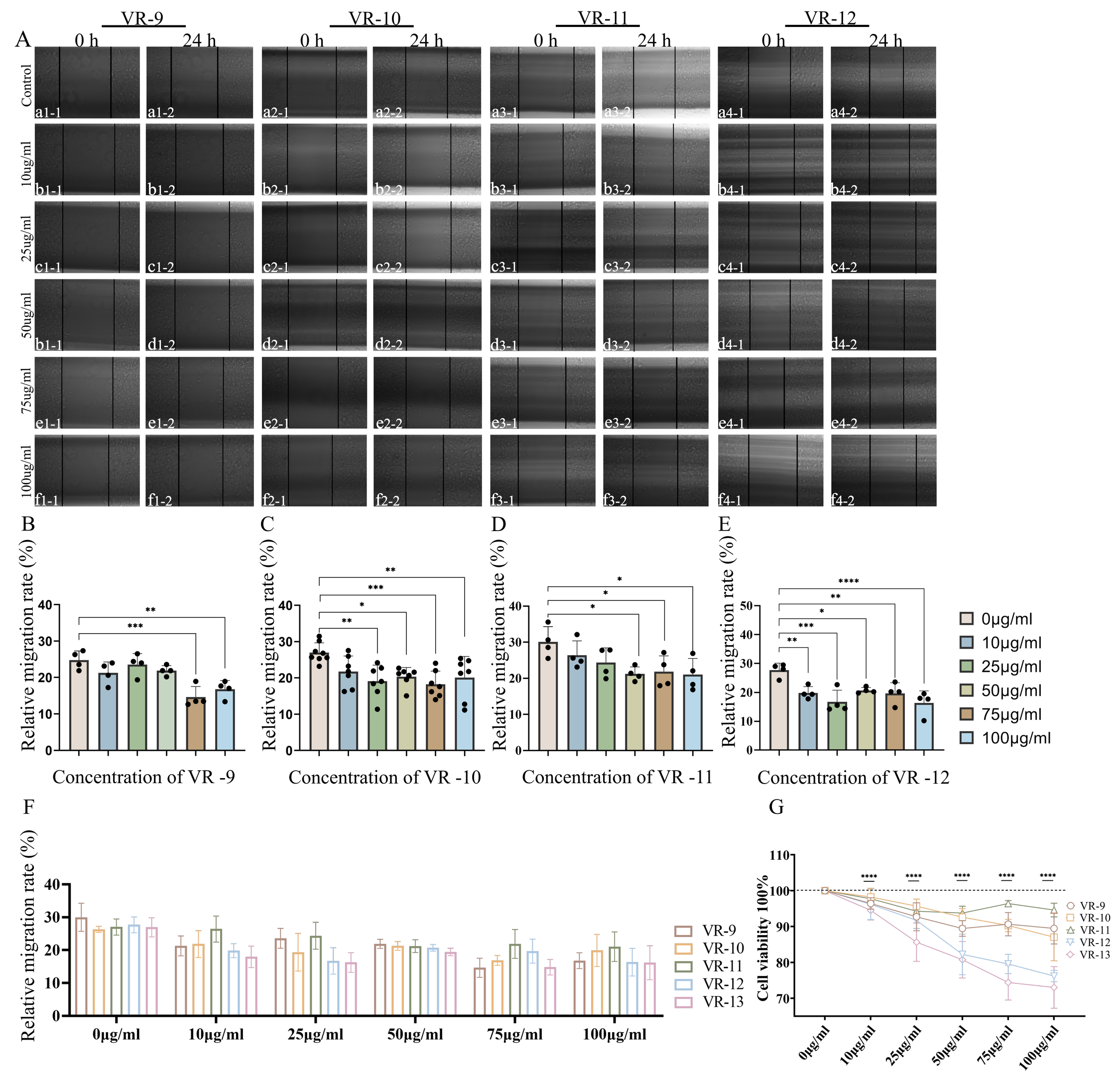

Supplement: S1 Fig — Wound-healing assay was used to detect the inhibitory effect of different concentrations of TSP-1 peptides (VR-9 to VR-12) on the proliferation and migration of RF/6A cells (a–e). VR-9 inhibited cell proliferation and migration at concentrations of 75 μg/mL and 100 μg/mL; VR-10 at concentrations of 25 μg/mL, 50 μg/mL, 75 μg/mL, and 100 μg/mL; VR-11 at concentrations of 50 μg/mL, 75 μg/mL, and 100 μg/mL; VR-12 inhibited cell proliferation and migration at all concentrations. The VR-13 inhibitory effect on cell proliferation and migration was the most significant among all TSP-1 peptide (f). CCK-8 assay showed that the inhibitory effect of each TSP-1 peptide on cell proliferation increased with increasing concentrations, with VR-13 showing the strongest inhibitory effect (g). (*, P < 0.05; **, P < 0.01; ***, P < 0.001; ****, P < 0.0001). (TIF) [file pone.0325661.s001.tif]

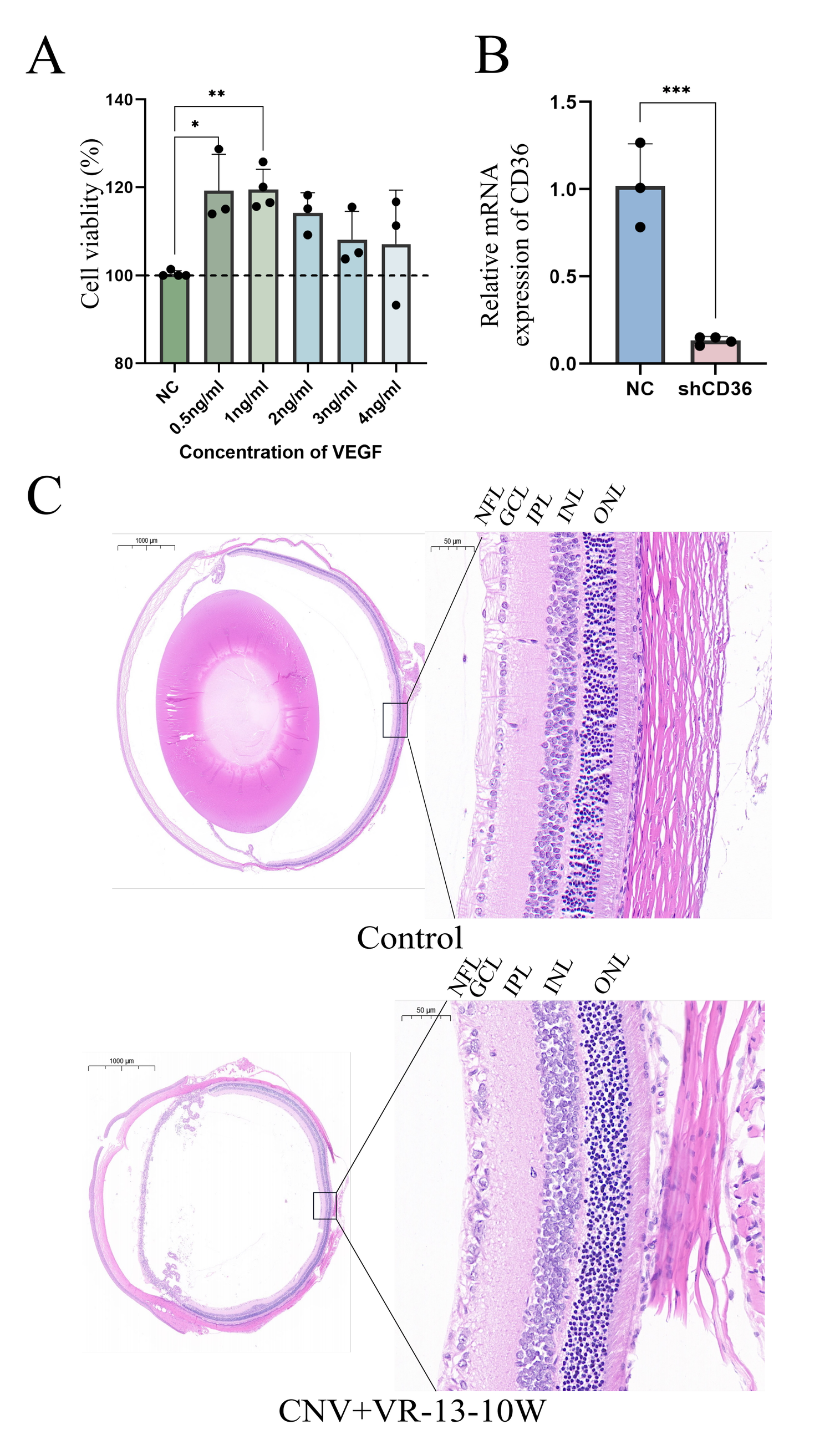

Supplement: S2 Fig — CCK-8 assay was used to detect the effect of different concentrations of recombinant human VEGF165 on the proliferation of RF/6A cells (a). CD36 knockdown verification by quantitative real-time polymerase chain reaction (qPCR) (b). Hematoxylin and eosin staining to observe the retinal architecture (c). (*, P < 0.05; **, P < 0.01; ***, P < 0.001; ****, P < 0.0001). (TIF) [file pone.0325661.s002.tif]
