# Supplementary material for: Active peptides of TSP-1 inhibit retinal angiogenesis through the CD36 pathway in a rat model of choroidal neovascularization
Source: PLoS One. 2025 Jun 20;20(6):e0325661. doi: 10.1371/journal.pone.0325661 (PMC12180637; doi:10.1371/journal.pone.0325661)
Supplement: S3 Fig — (PDF) [file pone.0325661.s004.pdf]

In order to simultaneously detect the target protein and internal reference on the same membrane, it is necessary to crop the membrane. The Marker used in this study was PageRuler Prestained Protein Ladder, 10 to 180 kDa (Thermo Fisher, THE-26616)

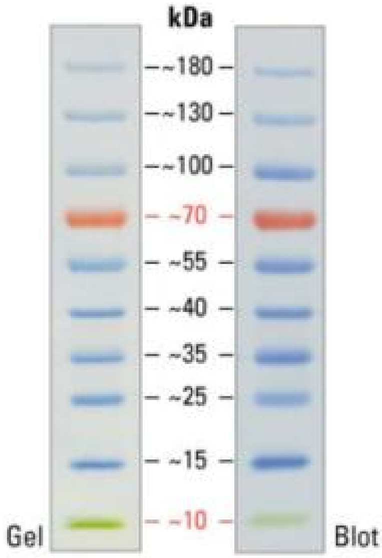

The molecular weight of the proteins involved in this study as follows:

Antibody	molecular weight（kda）
CD31	83
VEGF	40
β-actin	42

Fig3

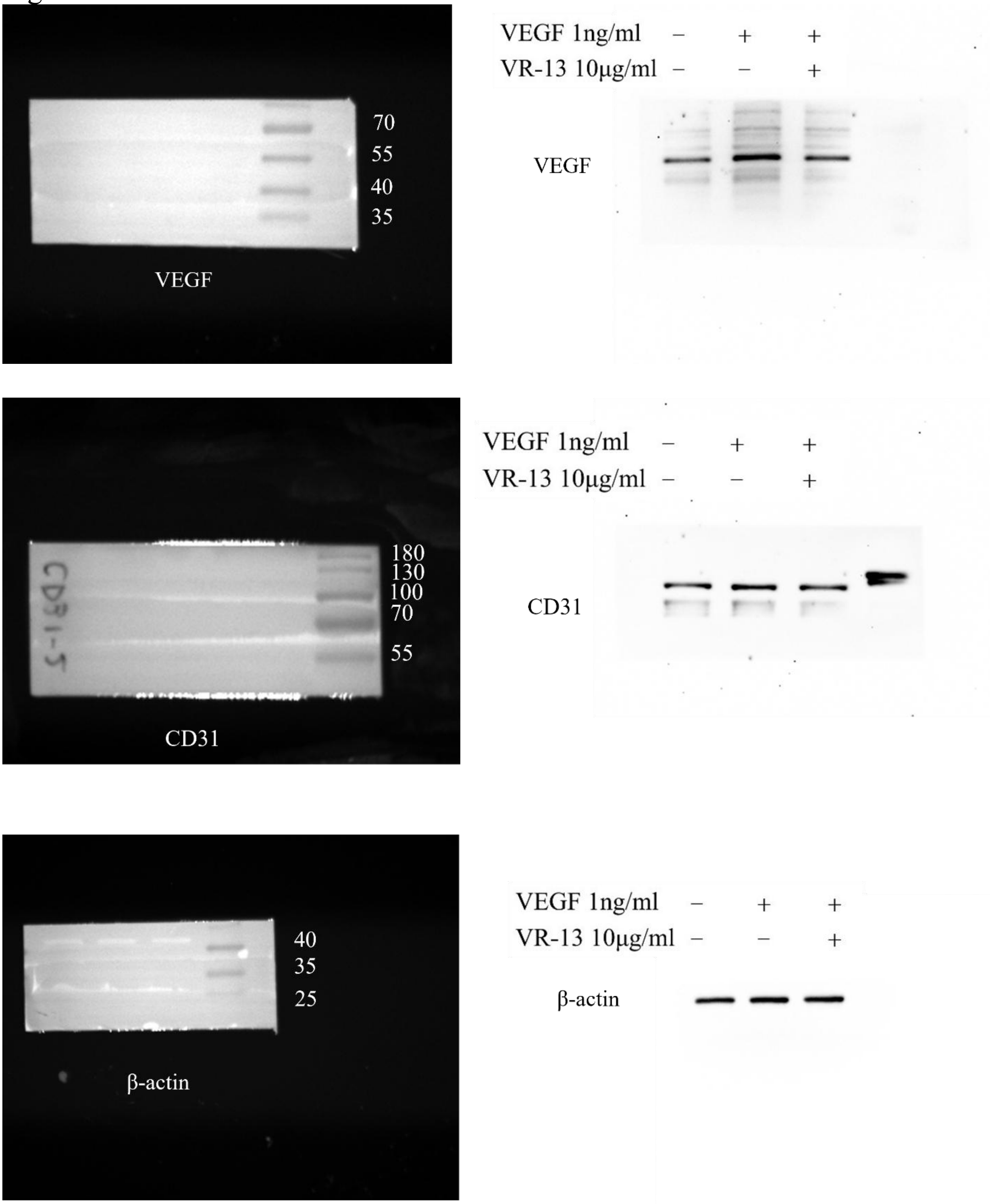
70
55
40
35
VEGF
VEGF 1ng/ml – + +
VR-13 10μg/ml – – +
VEGF
CD31-5
180
130
100
70
55
CD31
VEGF 1ng/ml – + +
VR-13 10μg/ml – – +
CD31
40
35
25
β-actin
VEGF 1ng/ml – + +
VR-13 10μg/ml – – +
β-actin

Fig4

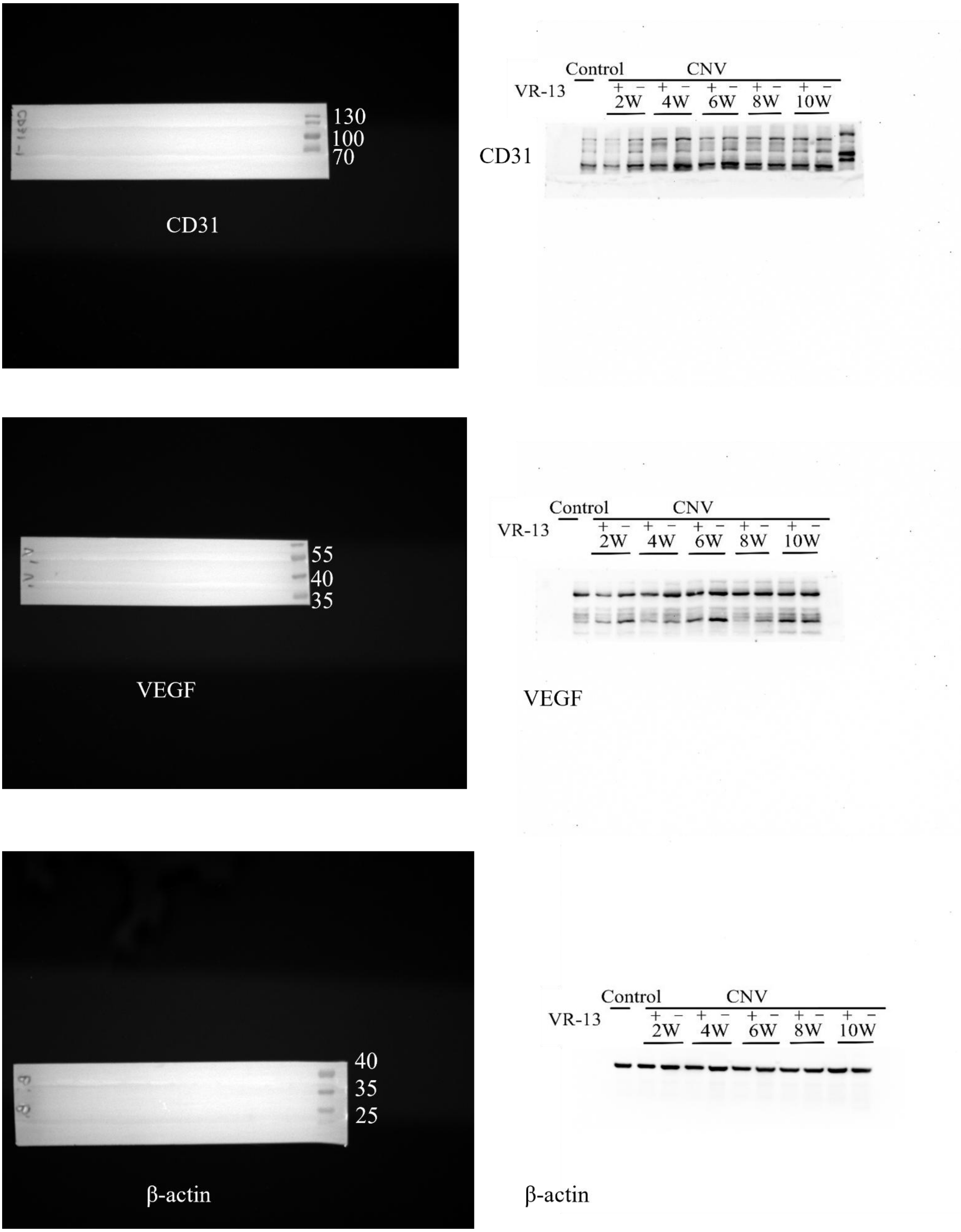
130
100
70
CD31
Control
CNV
VR-13
+ − + − + − + − + −
2W 4W 6W 8W 10W
CD31
55
40
35
VEGF
Control
CNV
VR-13
+ − + − + − + − + −
2W 4W 6W 8W 10W
VEGF
40
35
25
β-actin
Control
CNV
VR-13
+ − + − + − + − + −
2W 4W 6W 8W 10W
β-actin

Fig5

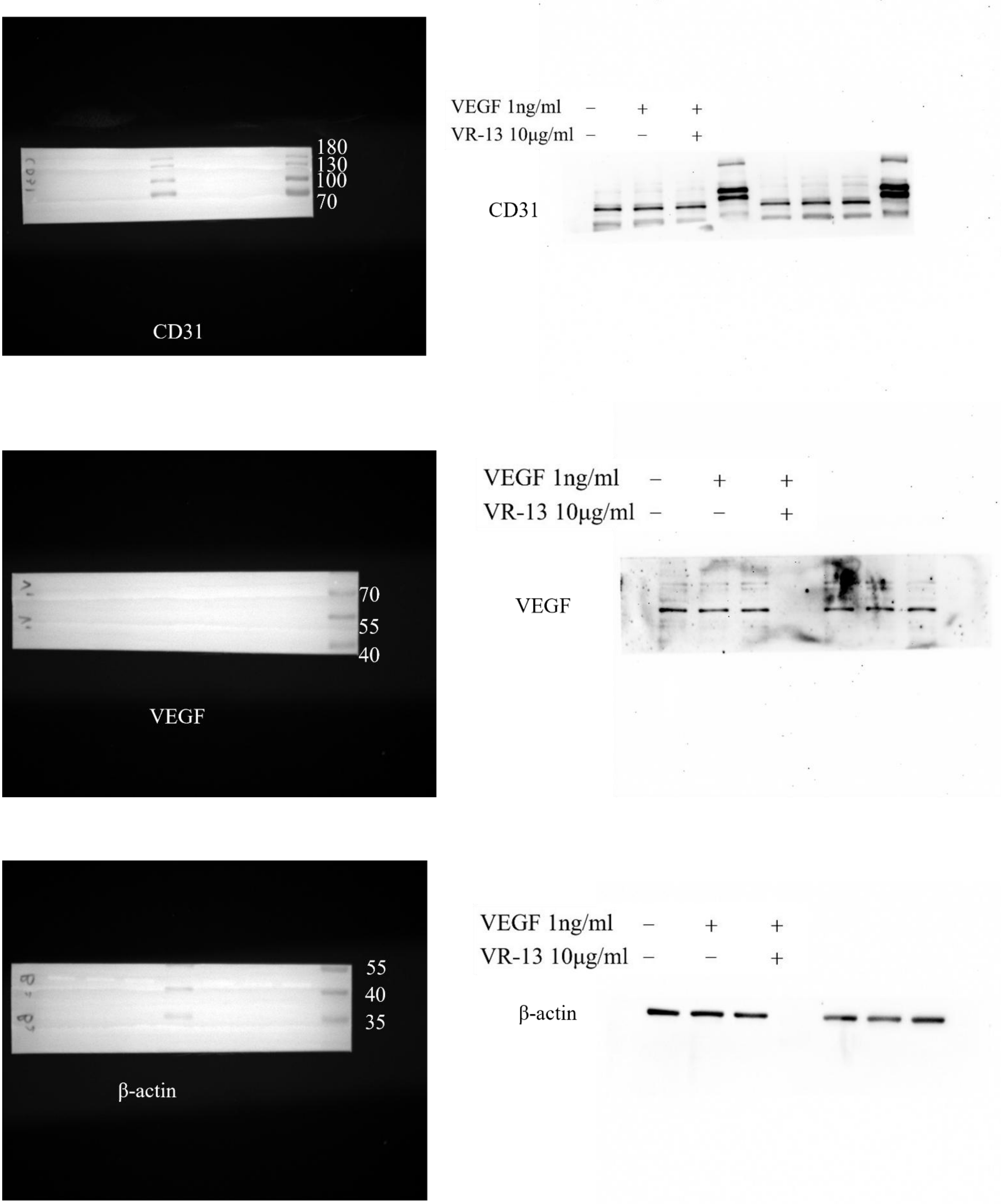
180
130
100
70
CD31
VEGF 1ng/ml − + +
VR-13 10μg/ml − − +
CD31
70
55
40
VEGF
VEGF 1ng/ml − + +
VR-13 10μg/ml − − +
VEGF
55
40
35
β-actin
VEGF 1ng/ml − + +
VR-13 10μg/ml − − +
β-actin
